# Supplementary material for: Antimicrobial resistant bacteria isolated from Buruli ulcer lesions in Ghana
Source: PLoS Negl Trop Dis. 2025 May 27;19(5):e0013140. doi: 10.1371/journal.pntd.0013140 (PMC12136463; doi:10.1371/journal.pntd.0013140)
Supplement: Attachment — Submitted filename: Response to Reviewers.docx [file pntd.0013140.s002.docx]

18th April 2025

**Kumasi Centre for Collaborative Research**

**Kwame Nkrumah University of Science and Technology**

**Kumasi, Ghana**

**The Editor**

**PLOS Neglected Tropical Diseases**

Dear Sir,

**Re: Antimicrobial resistant bacteria isolated from Buruli ulcer lesions in Ghana (PNTD-D-25-00354)**

My co-authors and I have taken note of the review comments and revised the manuscript as requested.

We wish to submit the revised manuscript for publication in your esteemed journal. In the attached reply, the reviewer questions are in blue font and our responses are in red font.

We look forward to the next steps towards the publication of our manuscript.

We count on your usual kind assurances.

Yours Sincerely,

Dr Yaw Ampem Amoako

Corresponding author

**Point by point response to review comments**
**Journal Requirements:**

Comment: 1) Some material included in your submission may be copyrighted. According to PLOSu2019s copyright policy, authors who use figures or other material (e.g., graphics, clipart, maps) from another author or copyright holder must demonstrate or obtain permission to publish this material under the Creative Commons Attribution 4.0 International (CC BY 4.0) License used by PLOS journals. Please closely review the details of PLOSu2019s copyright requirements here: PLOS Licenses and Copyright. If you need to request permissions from a copyright holder, you may use PLOS's Copyright Content Permission form.
Please respond directly to this email and provide any known details concerning your material's license terms and permissions required for reuse, even if you have not yet obtained copyright permissions or are unsure of your material's copyright compatibility. Once you have responded and addressed all other outstanding technical requirements, you may resubmit your manuscript within Editorial Manager. 
Potential Copyright Issues:
i) Figure 1. Please (a) provide a direct link to the base layer of the map (i.e., the country or region border shape) and ensure this is also included in the figure legend; and (b) provide a link to the terms of use / license information for the base layer image or shapefile. We cannot publish proprietary or copyrighted maps (e.g. Google Maps, Mapquest) and the terms of use for your map base layer must be compatible with our CC BY 4.0 license.
Note: if you created the map in a software program like R or ArcGIS, please locate and indicate the source of the basemap shapefile onto which data has been plotted.
If your map was obtained from a copyrighted source please amend the figure so that the base map used is from an openly available source. Alternatively, please provide explicit written permission from the copyright holder granting you the right to publish the material under our CC BY 4.0 license.
If you are unsure whether you can use a map or not, please do reach out and we will be able to help you. The following websites are good examples of where you can source open access or public domain maps:
* U.S. Geological Survey (USGS) - All maps are in the public domain. (http://www.usgs.gov)
* PlaniGlobe - All maps are published under a Creative Commons license so please cite u201cPlaniGlobe, http://www.planiglobe.com, CC BY 2.0u201d in the image credit after the caption. (http://www.planiglobe.com/?lang=enl)
* Natural Earth - All maps are public domain. (http://www.naturalearthdata.com/about/terms-of-use/).

Response: This map was generated using the OpenStreetMap on QGIS. OpenStreetMap® is open data, licensed under the Open Data Commons Open Database License (ODbL) by the OpenStreetMap Foundation (OSMF) (<https://www.openstreetmap.org> , CC BY-SA 2.0). The figure 1 legend has been modified to indicate the source of the basemap shapefile (line 156).
Comment: 2) We note that your Data Availability Statement is currently as follows: "All relevant data have been included in the manuscript.". Please confirm at this time whether or not your submission contains all raw data required to replicate the results of your study. Authors must share the “minimal data set” for their submission. PLOS defines the minimal data set to consist of the data required to replicate all study findings reported in the article, as well as related metadata and methods (https://journals.plos.org/plosone/s/data-availability#loc-minimal-data-set-definition).
For example, authors should submit the following data: 
1) The values behind the means, standard deviations and other measures reported;
2) The values used to build graphs;
3) The points extracted from images for analysis..
Authors do not need to submit their entire data set if only a portion of the data was used in the reported study.
If your submission does not contain these data, please either upload them as Supporting Information files or deposit them to a stable, public repository and provide us with the relevant URLs, DOIs, or accession numbers. For a list of recommended repositories, please see https://journals.plos.org/plosone/s/recommended-repositories. 
If there are ethical or legal restrictions on sharing a de-identified data set, please explain them in detail (e.g., data contain potentially sensitive information, data are owned by a third-party organization, etc.) and who has imposed them (e.g., an ethics committee). Please also provide contact information for a data access committee, ethics committee, or other institutional body to which data requests may be sent. If data are owned by a third party, please indicate how others may request data access.

Response: We confirm that the submission contains all raw data required to replicate the results of the study. ‘All relevant data have been included in the manuscript.’

Comment: 3) Please amend your detailed Financial Disclosure statement. This is published with the article. It must therefore be completed in full sentences and contain the exact wording you wish to be published.
1) State the initials, alongside each funding source, of each author to receive each grant. For example: "This work was supported by the National Institutes of Health (####### to AM; ###### to CJ) and the National Science Foundation (###### to AM).".
Response: Done as suggested. The section now reads:

‘This work was funded on the BuruliNox study which is part of the EDCTP2 programme supported by the European Union (101897 BuruliNox TMA 2016 SF-1509 to ROP) and the Deutsche Forschungsgemeinschaft (DFG; Project number: 461611374 to TT, DD, YAA and ROP). The funders had no role in study design; in the collection, analysis and interpretation of data; in the writing of the report; and in the decision to submit the article for publication.’

**Reviewers' Comments:**
Reviewer #1: Methods do not entirely cover what is needed to fully address the objectives of the study. Although the Introduction seems to promise a lot of novel information on the subject - the role of 'secondary infection' or perhaps, rather - the role of bacterial colonisation of BU wounds - on healing (i.e., impaired or delayed healing) of such wounds; - the last sentence of the Introduction Section seems to be much more modest i.e., describing colonisaton with potentially pathogeneic bacteria that MIGHT delay or impair BU wound healing. The authors provide a nice review of the literature on this subject; it remains unclear however, what information they plan to add to the existing literature. The Introduction promises a confirmation that bacterial secondary infection drives / causes delayed wound healing; but obviously this hypothesis cannot be proved by providing associations between delayed healing and bacterial colonisation; cause and effect cannot be proven using such methodology, without a prospective, well powered randomized intervention, associations do not prove cause and effect (ref 17 shows association, not causation). An interesting aspect of the study was that culture samples were obtained at three different points over time. One weakness is obviously, the fact that only isolates were obtained by culture; if genomic methods had been used with quantification, perhaps a much more realistic reflection of potential bacterial pathogens would have been generated.
Even if that be the case, the methods are not entirely clear;
1) although selection criteria were described for study participants, no sample size estimation was provided; was it rather a convenience sample? Why stop enrolling after 56 participants were enrolled? Could the authors provide a flow chart for readers to understand how many screened patients were excluded, and for what reasons?

Response: It is an increasingly recognised fact that the number of Buruli ulcer cases are on the decline in west Africa. Thus all patients who were diagnosed during the study period who met the inclusion criteria and provided informed consent were recruited into the study. We have revised the section to indicate that a convenient sampling technique was used. The section now reads:

‘Participants were selected using a convenience sampling technique.’ (line 150)

2) how exactly did they swab the lesions? Amissah et al used a strictly defined method to sample the whole surface of lesions using the bandages of lesions removed during wound care and dressing changes; other study groups sampled by swab using a less clearly defined method, obviously introducing noise and perhaps even, bias in the study methodology; please explain the sampling in more detail.

Response: We have indicated that swabs were taken from the surface of lesions using the Levine method. The section now reads:

‘At each sampling time, sterile swab sticks moistened with phosphate-buffered saline were used to swab the surface of the ulcer lesions following the Levine sample collection method [29]. Briefly, samples were collected by rotating the tip of the sterile swab over a 1 cm^2^ area of wound surface while exerting sufficient pressure to extract fluid from the wound tissue. For nodules, oedematous and plaque lesions that had began to ulcerate, samples were collected from the ulcerated surfaces of these lesions.’ (line 167-172)

3) although most lesions were ulcers or plaque, it puzzeled this reviewer how the authors sampled nodules and edematous lesions; or did these lesions break down into ulcers later on, and were the samples only taken after the lesions broke down into ulcers?

Response: We included a footnote in Table 1 to indicate that ‘nodules, oedematous and plaque lesions had began to ulcerate. Samples were collected from the ulcerated surfaces of these lesions. We have now moved this to the section on laboratory procedures. (line 171-172)

Reviewer #3: Study design was appropriate but there are errors/omissions in the statistical analysis as outlined below.

Response: The responses to specific comments by this reviewer are detailed in the relevant sections below.

Results

Reviewer #1: The Results summarize the findings obtained from the 56 study participants, with a total of 196 isolates of bacterial species and their antimicrobial resistance using EUCAST criteria for breakpoints, with most isolates obtained at baseline; 76 isolates at baseline, 43 at week 8 and 50 at week 16. Although table 1 summarizes the data on the study subjects, many different aspects that have potential impact on wound healing are not provided; nutritional status, duration of disease, exact lesional size etc are not presented; the analysis is straightforward but simple; no attempts were made to corroborate the (flawed) opinion of the authors that co-infection or colonisation with these potential pathogens impair wound healing; e.g., no logistic regression models were provided to suggest that harbouring these pathogens was associated with delayed or impaired wound healing.
For this reviewer, it remains unclear whether positive cultures were obtained in specific patient groups; and how many turned out to have a negative culture result. We only see the results of cultures, no breakdown with study participants as the denominator .

Response: Lesion size in Buruli ulcer disease is assessed using the WHO lesion categories; this information was provided in Table 1.

We did not collect information on nutritional status as this was not the focus of the current study; however, our group has previously published on the role of nutrition in BU disease (Loglo AD, et al. Micronutrient-deficient diets and possible environmental enteric dysfunction in Buruli ulcer endemic communities in Ghana: Lower dietary diversity and reduced serum zinc and vitamin C implicate micronutrient status a possible susceptibility factor. PLoS Negl Trop Dis. 2025 Mar 12;19(3):e0012871).

Several factors impact healing including presence of viable mycobacteria, lesion category, development of Paradoxical Reactions etc (*Frimpong M, et al. Paradoxical reactions in Buruli ulcer after initiation of antibiotic therapy: Relationship to bacterial load. PLoS Negl Trop Dis. 2019;13(8):e0007689; Agbavor B, et al. Clinical and microbiological predictors of healing in Buruli ulcer disease. J Clin Tuberc Other Mycobact Dis. 2024;34:100415*). Yet, the presence of pathogenic organisms in any wound is concerning as these organisms can impact healing irrespective of other factors.

We have included a flow chart (figure 2) to indicate how many patients were sampled at each time point and how many resulted in positive cultures.

Conclusions
Reviewer #1: The Conclusion itself is supported by the data provided.
In The Discussion section, many aspects are discussed that were actually not addressed by the study team; the mentioning that drug-resistant pathogens are a threat to the health care system may be true, but this seems speculative as this aspects was not covered in the analysis. Their mentioning and discussing antimicrobial stewardship is therefore a bit of a long shot . . One might argue that stricter adherence to appropriate wound care could suffice; others have demonstrated (using Whole Genome Sequencing) that in-hospital transmission occurred, at least with MRSA; the authors themselves have not provided added information to corroborate this.

Response: We are happy to note the reviewer’s agreement with the statement that drug resistant pathogens are a threat to the health care system. These patients with Buruli ulcer disease receive wound care within healthcare settings and in their homes in Ghana and so the presence of such resistant organisms within such care settings is concerning. We did not perform Whole Genome Sequencing which may have indicated if in-hospital transmission occurred and this has been acknowledged as a limitation of the current study (line 391-393).

 Editorial and Data Presentation Modifications?
Reviewer #1:

Comment: The work represents a confirmation of earlier work by other groups that indeed, potential bacterial drug-resistant pathogens can be isolated from Buruli ulcer lesions; the data set is not extremely rich, and lacks sophisticated data analysis that would potentially support existing hypotheses, notably, the hypothesis that these organisms impair wound healing in BU. A flow chart and a per-study-participant table would help the reader to appreciate the work more meaningfully.

Response: We have included a flow chart as suggested (figure 2) to indicate how many patients were sampled at each time point and how many resulted in positive cultures.

Reviewer #2: Minor Revision

Response: Thank you

Reviewer #3: Minor modifications:
Comment - Line 155: Why were pregnant or breast-feeding women excluded from the study?

Response: Pregnant and breastfeeding women with BU disease are not treated with rifampicin and clarithromycin (but are offered surgical resection of the lesion). Thus such individuals could not be included in a study that seeks to assess the patterns of antimicrobial resistance in pathogenic bacterial isolates from BU lesions before and during antibiotic treatment.
Comment - Line 197: suggest change to Among these, 33 (59%) were males as no need to repeat 33/56

Response: Done as suggested
Comment - Line 241: None of the increases are statistically significant (p>0.05). This should be clarified.

Response: We have modified the section appropriately and it now reads: ‘At week 16, a higher proportion of *Klebsiella* spp. (1/2, 50%), *P. mirabilis* (9/10, 90%) and *P. stuartii* (3/4, 75%) showed resistance to trimethoprim/sulfamethoxazole compared to week 8.’ (line 252-254)
Comment - Line 251: need to clarify that the stats provided here for tigecycline and nitrofurantoin are for resistance, as it reads from earlier in the sentence that they are susceptible.

Response: The section has been reviewed and now reads: ‘Among Gram-positive bacteria, there was no resistance of *E. faecalis* isolates to all tested antibiotics except tigecycline (1/19, 5.3%) and nitrofurantoin (2/19, 10.5%) at baseline.’ (line 262-263)
Comment - Line 262: None of the increases are statistically significant (p>0.05). This should be clarified.

Response: Th section has been modified and now reads: ‘However, a higher proportion of the bacterial isolates at week 16 exhibited resistance to the majority of the selected antibiotics, including oxacillin (9/15, 60%), tetracycline (14/15, 93.3%) and rifampicin (2/2, 100%) compared to before treatment.’
Comment - Line 275: In table 3 oxacillin sensitivities are not included, yet reported in Line 262.

Response: The sensitivities to oxacillin have been included in Table 3.
Comment - Line 292: suggest modify to “insights into the local resistance patterns associated with BU in Ghana…”

Response: Done as suggested (line 296)
Comment - Line 294: suggested modify to “was slightly higher than previously reported.”

Response: Done as suggested (line 298)
Comment - Line 295: majority of patients were not <15 years (only 39.3%)

Response: The section has been revised appropriately by deleting the sentence.
Comment - Line 322: suggest modify to “, along with the provision…”

Response: Done (line 325)
Comment - Line 340: suggest change to “were observed against tetracyclines”

Response: Done (line 343)
Comment- Line 343: suggest change to “After treatment (week 16)…”

Response: Done (line 346)

Summary and General Comments

Reviewer #1: Strengths: sample size is larger than their earlier study, with a nice longitudinal design with sampling at three different time points;
Novelty: confirmatory, not novel;
Significance: not entirely clear, as we already know that drug-resistant bacterial isolates can be abundantly recovered from BU lesions; we also know that current practice to start simple antimicrobials for suspected secondary infection is often inappropriate and potentially harmful; we still don't know whether culture-based targeted therapy brings any good for patients, and whether wound healing would be improved; only a controlled clinical trial would be able to provide that answer, or if that is unfeasible, perhaps, a data-rich, large clinical database of considerable size (c.f., the Individual Patient Data analysis that has been used in MDRTB) could be used to conduct a sophisticated multivariable analysis to provide circumstantial evidence to support the hypothesis that such therapy might be beneficial.
Weaknesses: culture-based data are difficult to interpret; quantitative genomic analysis might be a much more appropriate study design, but obviously this might be unaffordable; sampling technique is critical, and the authors should either exactly explain the sampling technique, or admit that leaving it to the discretion of the attending study team member on the ground might introduce noise, or even bias.

Response: Many thanks for these comments. We agree with the reviewer that genomic quantitative analysis may have yielded additional information on the subject matter; however, we did not have sufficient funding to enable us undertake whole genome sequencing (WGS). Additionally, culture based methods are more widely available and remain a valuable tool for studying antimicrobial resistance in limited resource settings where facilities for performing WGS are limited.

We have indicated that swabs were taken from the surface of lesions using the Levine method. The section now reads: ‘At each sampling time, sterile swab sticks moistened with phosphate-buffered saline were used to swab the surface of the ulcer lesions following the Levine sample collection method [29]. Briefly, samples were collected by rotating the tip of the sterile swab over a 1 cm^2^ area of wound surface while exerting sufficient pressure to extract fluid from the wound tissue. For nodules, oedematous and plaque lesions that had began to ulcerate, samples were collected from the ulcerated surfaces of these lesions.’ (line 167-172)

Reviewer #2: Abstract
• Background: there are some redundancy with “antimicrobial resistance”

Response: We have deleted the word ‘patterns’. The section now reads: ‘Despite this, there is limited data on the antimicrobial resistance of these bacterial isolates within BU lesions.’
• Methodology: “ESBL production” can you provide the definition of “ESB;” as is the first apparition on the manuscript. Same for “MRSA” and “MDR”
Response: Done as suggested
Methods
• Improved the presentation of tables 2 and 3. reading and understanding are not always easy
Response: We are unsure what exact changes the reviewer wants to see. The tables represents the resistance profile of Gram negative and Gram positive bacterial isolates. We indicated that numerators represent number of isolates that were resistant to a given antibiotic and denominators represent the total number of isolates tested at the time point. The Tables are unchanged.

Discussion
• Line 304 “Yeboah-Manu et al” must be written in italics and the same applies to the entire document
Response: We do not think that using italics in this case is necessary. We have chosen to stick to the standard approach.
General comments:
• Quality of the images should be ameliorated
• Use the same police to presented the references

Response: The figures in this manuscript were PACE corrected in line with the journal standards.

Reviewer #3: In general, an interesting paper to demonstrate the type and prevalence and the antibiotic sensitivities of bacterial colonising flora of BU wounds pre, during and post BU treatment in Ghana.
However, I think that there are some points that would be important to address as follows:
- It is important that the reader understands that the type and prevalence of organisms and their antibiotic sensitivities may vary depending on location. Thus the organisms isolated in this study are reflective of the situation in regional Ghana but not necessarily reflective of what may be found in other settings (as recognised in line 307). It should be mentioned therefore that when advocating for this information to be utilised to develop treatment guidelines, it should be recognised that to maximise usefulness, local settings will need to do their own surveillance microbiological data.

Response: Many thanks for the comment. We have modified the conclusion section to address the review concern and it now reads:

‘There is a concerning prevalence of antimicrobial resistant bacteria, including MDR, ESBL-positive and MRSA pathogens, in Buruli ulcer lesions. These findings underscore the urgent need for the development of integrated guidelines to guide surveillance and treatment of secondary bacterial infections to further improve outcomes in BU. To maximise usefulness, local settings will need to conduct surveillance to provide appropriate microbiological data to support the development of local antibiotic guidelines.’

Comment - It is regularly stated in the paper (eg lines 282, 313, 362, 367) that there was an increase in MRSA from baseline to Wk 16 – however the increase is not statistically significant and may just represent chance, especially with the small sample size (6/12 v 9/15, p-value >> 0.05). These statements need to be modified to reflect this.

Response: Done as suggested. We have also added to the limitation section the following sentences:

‘Although we observed an increase in the proportion of pathogenic organisms such as MRSA and *Proteus mirabilis* that were resistant to the tested antibiotics, the overall numbers were relatively low. Thus, similar studies in other endemic settings involving larger number of participants are needed to corroborate these findings.’
Comment - Likewise in line 220 the increase in P. mirabilis from 7/43 to 10/50 is not statistically significant (p>>0.05), and in Line 281 the decrease in ESBLs does not reach statistical significance.

Response: Done as suggested. We have also added to the limitation section the following sentences:

‘Although we observed an increase in the proportion of pathogenic organisms such as MRSA and *Proteus mirabilis* that were resistant to the tested antibiotics, the overall numbers were relatively low. Thus, similar studies in other endemic settings involving larger number of participants are needed to corroborate these findings.’ (line 400-403)
Comment - In table 3, why were only 2 isolates tested for rifampicin – it is important, especially on treatment, to understand if there is any increase in resistance in colonizing flora to the antibiotics used in treating BU (ie rifampicin and clarithromycin). This is important AMR information. Further to this it would be worth noting the results from table 3 that show no significant increase in macrolide resistance in S. aureus isolates on treatment.

Response: The low proportion of isolates tested for rifampicin resistance was due to logistical constraints.

We have also added the following sentences to the results section:

‘The proportion of *S aureus* isolates that showed resistance to macrolides like erythromycin during treatment was relatively low. One of two (50%) *S aureus* isolates were resistant to rifampicin at week 0 while 2/2 (100%) were resistant at week 16.’ (line 275-278)
Comment- Interesting to note in Line 219 that the proportion of S. aureus increased and this is statistically significant (P<0.05) which would be worth mentioning. This is a little unexpected in view of rifampicin and clarithromycin’s usual anti-staph activity – maybe worth further explaining this fact in line 316 of discussion. However I think it is important to consider that it may also be due to the induction of resistance in these organisms (especially to rifampicin as not to clarithro according to table 3). Thus a bit more discussion about what the AMR implications for S. aureus are with current BU treatments would be important.

Response: Done as suggested (line 331-334)
