# Supplementary material for: Antimicrobial resistant bacteria isolated from Buruli ulcer lesions in Ghana
Source: PLoS Negl Trop Dis. 2025 May 27;19(5):e0013140. doi: 10.1371/journal.pntd.0013140 (PMC12136463; doi:10.1371/journal.pntd.0013140)
Supplement: Attachment — Submitted filename: Response_to_Reviewers_auresp_2.docx [file pntd.0013140.s003.docx]

29th April 2025

**Kumasi Centre for Collaborative Research**

**Kwame Nkrumah University of Science and Technology**

**Kumasi, Ghana**

**The Editor**

**PLOS Neglected Tropical Diseases**

Dear Sir,

**Re: Decision on your PLOS Neglected Tropical Diseases submission (PNTD-D-25-00354R1) - [EMID:50ace321a419cfe0]**
My co-authors and I have taken note of the review comments and revised the manuscript as requested.

We wish to submit the revised manuscript for publication in your esteemed journal. In the attached reply, the reviewer questions are in blue font and our responses are in red font.

We look forward to the next steps towards the publication of our manuscript.

We count on your usual kind assurances.

Yours Sincerely,

Dr Yaw Ampem Amoako

Corresponding author

**Point by point response to review comments**

**Additional Editor Comments:**

Dear Amoako and colleagues,
Your revised version appears to adequately answer the reviewers' concerns. However, you did not include Figure 1. Please provide the figure to the journal, assuming that it now meets all specifications.
Thank you for submitting your work to PLoS Neglected Tropical Diseases.

Response: Thank you for this comment. We have now uploaded Figure 1 to the online submission system.

**Journal Requirements:**

Please upload a copy of Figure Figure 1 which you refer to in your text on page 6 and 7. Or, if the figure is no longer to be included as part of the submission please remove all reference to it within the text.
Response: Thank you. We have uploaded Figure 1 to the online submission system.
